# Therapeutic efficacy of dihydroartemisinin-piperaquine combination for the treatment of uncomplicated malaria in Ghana

**DOI:** 10.3389/fcimb.2022.1058660

**Published:** 2023-01-06

**Authors:** Benjamin Abuaku, Paul Boateng, Nana Yaw Peprah, Alexander Asamoah, Nancy Odurowah Duah-Quashie, Sena Adzoa Matrevi, Eunice Obeng Amoako, Neils Quashie, Felicia Owusu-Antwi, Keziah Laurencia Malm, Kwadwo Ansah Koram

**Affiliations:** ^1^ Department of Epidemiology, Noguchi Memorial Institute for Medical Research, College of Health Sciences, University of Ghana, Legon, Accra, Ghana; ^2^ National Malaria Elimination Program, Public Health Division, Ghana Health Service, Accra, Ghana; ^3^ Centre for Tropical Clinical Pharmacology and Therapeutics, University of Ghana Medical School, Accra, Ghana; ^4^ Country Office, World Health Organization, Accra, Ghana

**Keywords:** efficacy, dihydroartemisinin-piperaquine, uncomplicated malaria, treatment, Ghana

## Abstract

In 2020, Dihydroartemisinin-Piperaquine (DHAP) was adopted as a second-line antimalarial for treatment of uncomplicated malaria in Ghana following a review of the country’s antimalarial medicines policy. Available data obtained in 2007 had shown PCR-uncorrected therapeutic efficacy of 93.3% using a 28-day follow-up schedule. In 2020, the standard 42-day follow-up schedule for DHAP was used to estimate efficacy levels among febrile children aged 6 months to 9 years in three malaria sentinel sites representing the three main ecological zones of the country- savannah, forest, and coastal. PCR genotyping distinguished between recrudescence and re-infection using merozoite surface protein 2 (MSP2)-specific primers for FC27 and 3D7 strains. Per protocol analyses showed day 28 efficacy of 100% in all three sentinel sites with day 42 PCR-corrected efficacy ranging between 90.3% (95% CI: 80.1 – 96.4%) in the savannah zone and 100% in the forest and coastal zones, yielding a national average of 97.0% (95% CI: 93.4 – 98.8). No day 3 parasitemia was observed in all three sites. Prevalence of measured fever (axillary temperature ≥ 37.5°C) declined from 50.0 - 98.8% on day 0 to 7.1-11.5% on day 1 whilst parasitemia declined from 100% on day 0 to 1.2 - 2.3% on day 1. Mean haemoglobin levels on days 28 and 42 were significantly higher than pre-treatment levels in all three sites. We conclude that DHAP is highly efficacious in the treatment of uncomplicated malaria in Ghana. This data will serve as baseline for subsequent DHAP efficacy studies in the country.

## 1 Introduction

Malaria remains one of the major public health problems globally, accounting for 241 million cases and 627,000 deaths in 2020 with Ghana contributing to 2.1% and 1.9% of the cases and deaths, respectively (WHO, 2021). In 2021, confirmed malaria cases accounted for 19.8% of all out-patient illnesses and 19.6% of all admissions in Ghana (GHS, 2022). Malaria parasite prevalence among children under 5 years has consistently decreased over the 5-year period of 2014 – 2019: from 27% to 14% (GSSICF, 2019).

In 2010 the World Health Organization (WHO) included Dihydroartemisinin-Piperaquine (DHAP) as an artemisinin-based combination therapy (ACT) option for the treatment of uncomplicated *Plasmodium falciparum* malaria (WHO, 2010). This decision was based on data from several studies conducted between 2002 and 2010, showing failure rates of less than 5% similar to artesunate-amodiaquine (ASAQ), artemether-lumefantrine (AL), and artesunate-mefloquine (AM) combinations (WHO, 2010; Zani et al., 2014). DHAP was also found to be safe with similar adverse events as AL, and showed longer prophylactic effect on new infections (Karema et al., 2006; Myint et al., 2007; Price and Douglas, 2009; WHO, 2010; Zani et al., 2014). The longer prophylactic effect of DHAP is attributed to piperaquine, which is a bisquinoline antimalarial with elimination half-life of 14 – 21 days, similar to mefloquine but longer than lumefantrine (1 – 10 days) and amodiaquine (4 – 10 days) (Kamya et al., 2007; Zani et al., 2014; Bretscher et al., 2020).

In Ghana, DHAP and AL were considered alternate ACTs for patients unable to tolerate ASAQ three years after the introduction of ASAQ as first-line drug for the treatment of uncomplicated malaria (MOH, 2009). At that time the day 28 PCR-uncorrected cure rate for DHAP was estimated as 93.3% (95% CI: 88.3-96.3) (Abuaku et al., 2014). There were no subsequent efficacy studies on DHAP until 2020 when the country’s antimalarial medicine policy was revised and DHAP made second-line ACT for the treatment of uncomplicated malaria whilst ASAQ and AL remained first-line ACTs (MOH, 2020). We report the therapeutic efficacy of DHAP in three of ten sentinel sites representing the three main ecological zones of the country between October 2020 and April 2021 using the WHO, 2009 protocol (WHO, 2009).

## 2 Materials and methods

### 2.1 Study sites

**Figure 1:**
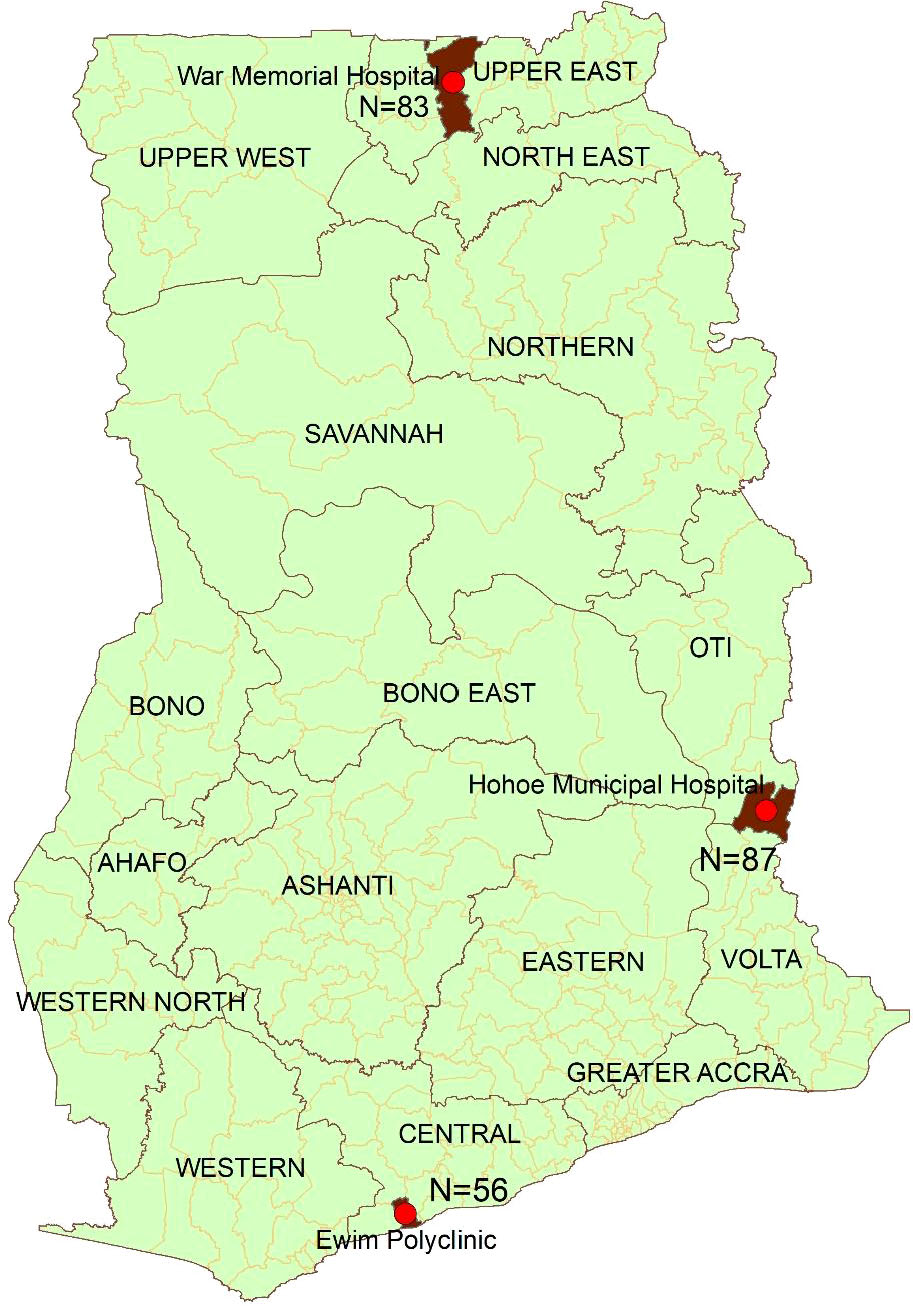
Map of Ghana showing the three sentinel sites as red dots.

The study was conducted in the War Memorial Hospital (WMH) in Navrongo (representing the savannah zone); Hohoe Municipal Hospital (HMH) in Hohoe (representing the forest zone); and Ewim polyclinic (EWP) in Cape-Coast (representing the coastal zone) (**Figure 1**). These sites have been described elsewhere (Abuaku et al., 2019). Briefly, malaria transmission in the savannah zone is perennial with marked seasonal variation. Malaria transmission in the forest zone is intense and perennial whilst transmission in the coastal zone is perennial but not intense (Abuaku et al., 2019).

### 2.2 Study design

The study was a one-arm prospective evaluation of the clinical, parasitological and hematological responses of children treated with DHAP in three of Ghana’s ten sentinel sites for monitoring therapeutic efficacy of first-line and second-line antimalarials.

### 2.3 Patient enrolment, treatment and follow-up

Children aged between 6 months and 9 years were included in this study, if they had a history of fever during the past 24 hours or an axillary temperature ≥ 37.5°C with mono *Plasmodium falciparum* infection and a parasite density ranging between 1,000 and 250,000 per µL. Children with severe malaria or whose parents refused to give consent were excluded from the study. As per the WHO protocol on methods for surveillance of antimalarial drug efficacy, a prior intake of an antimalarial was not an exclusion criterion (WHO, 2009).

Following parental consent, enrolled children were treated with 20 mg/160 mg or 40 mg/320 mg of D-ARTEPP^®^ (products of Guilin Pharmaceutical Company Limited, Guangxi, China) based on their weight in kilograms as per the manufacturer’s instructions (**Table 1**). All children were observed for 30 minutes after drug administration. Those who vomited within the 30 minutes after treatment received a repeated dose of D-ARTEPP^®^. Children with repeated vomiting were withdrawn from the study and treated as severe malaria cases as per national case management guidelines (MOH, 2020).

**Table 1:** Treatment doses for Dihydroartemisinin-piperaquine (DHAP) combination.

Weight (Kg)	Dihydroartemisinin- Piperaquine base	Total dose	Day		
			0	1	2
From 5Kg to Less than 8Kg (≥ 5Kg to < 8Kg)	20mg/160mg	3 tablets	1	1	1
From 8Kg to Less than 11Kg (≥ 8Kg to < 11Kg)	20mg/160mg	4 ½ tablets	1 ½	1 ½	1 ½
From 11Kg to Less than 17Kg (≥ 11Kg to < 17Kg)	40mg/320mg	3 tablets	1	1	1
From 17Kg to Less than 25Kg (≥ 17Kg to < 25Kg)	40mg/320mg	4 ½ tablets	1 ½	1 ½	1 ½
From 25Kg to Less than 36Kg (≥ 25Kg to < 36Kg)	40mg/320mg	6 tablets	2	2	2

Each child was followed-up for a period of 42 days. The follow-up schedule involved clinical assessment (days 0, 1, 2, 3, 7, 14, 21, 28, 35, 42, and any unscheduled visit within the 42-day follow-up period); drug administration (days 0, 1, 2); parasitological examination (days 0, 2, 3, 7, 14, 21, 28, 35, 42, and any unscheduled visit within the 42-day follow-up period); and hemoglobin level assessment (days 0, 28, and 42) using an automated hematology analyser (Mindray BC-2800™).

All malaria blood slides for parasitological examination were examined by two microscopists. Slides with discordant readings were re-examined by a third senior microscopist. Discordant readings were related to presence/absence of asexual/sexual parasites, species diagnosis, and day 0 parasite density within range of inclusion criterion (1,000 – 250,000 per µL). Parasite recrudescence was distinguished from re-infection by PCR genotyping using merozoite surface protein 2 (MSP2)-specific primers for FC 27 and 3D7 strains. List of specific sequence of primers used have been published elsewhere (Duah et al., 2016). Samples with post-treatment alleles having the same band sizes as pre-treatment alleles (base pairs) were classified as recrudescence (WHO, 2007).

### 2.4 Data analysis

A minimum sample of 55 children was estimated for each study site based on 5% PCR-corrected treatment failure rate at 95% confidence level, 6% precision, and10% loss to follow-up. Data for each child was captured using the WHO Excel^®^ template for therapeutic efficacy tests. The primary study outcomes were day 3 parasitemia and days 28 and 42 PCR-uncorrected and PCR-corrected efficacy outcomes. Day 3 parasitemia was evaluated as the proportion of patients seen on day 3 with parasitemia. Per protocol and Kaplan Meier survival analyses were used to describe the patterns of PCR-uncorrected and PCR-corrected treatment outcomes on days 28 and 42 post-treatment. Treatment outcomes analyzed were defined as early treatment failure (ETF), late parasitological failure (LPF), late clinical failure (LCF), and adequate clinical and parasitological response (ACPR) as per WHO protocol (WHO, 2009). Secondary study outcomes were measured fever (axillary temperature ≥ 37.5°C), parasite clearance, changes in mean hemoglobin levels, and prevalence of adverse events. Proportions were compared using Chi-square and Fisher’s exact tests whilst means were compared using student t-test/ANOVA (significant at p<0.05).

### 2.5 Ethics approval and consent to participate

The Institutional Review Board (IRB) of the Noguchi Memorial Institute for Medical Research, University of Ghana (FWA 00001824) approved this study (NMIMR-IRB CPN 032/05-06a amend. 2020). Written informed consent was obtained from each parent/guardian prior to commencement of the study. Each parent/guardian was presented with details of the study: objectives, methods, anticipated risks and benefits. They were also informed of their right to withdraw their children from the study at anytime during the study period without penalty.

## 3 Results

### 3.1 Patient characteristics

**Figure 2:**
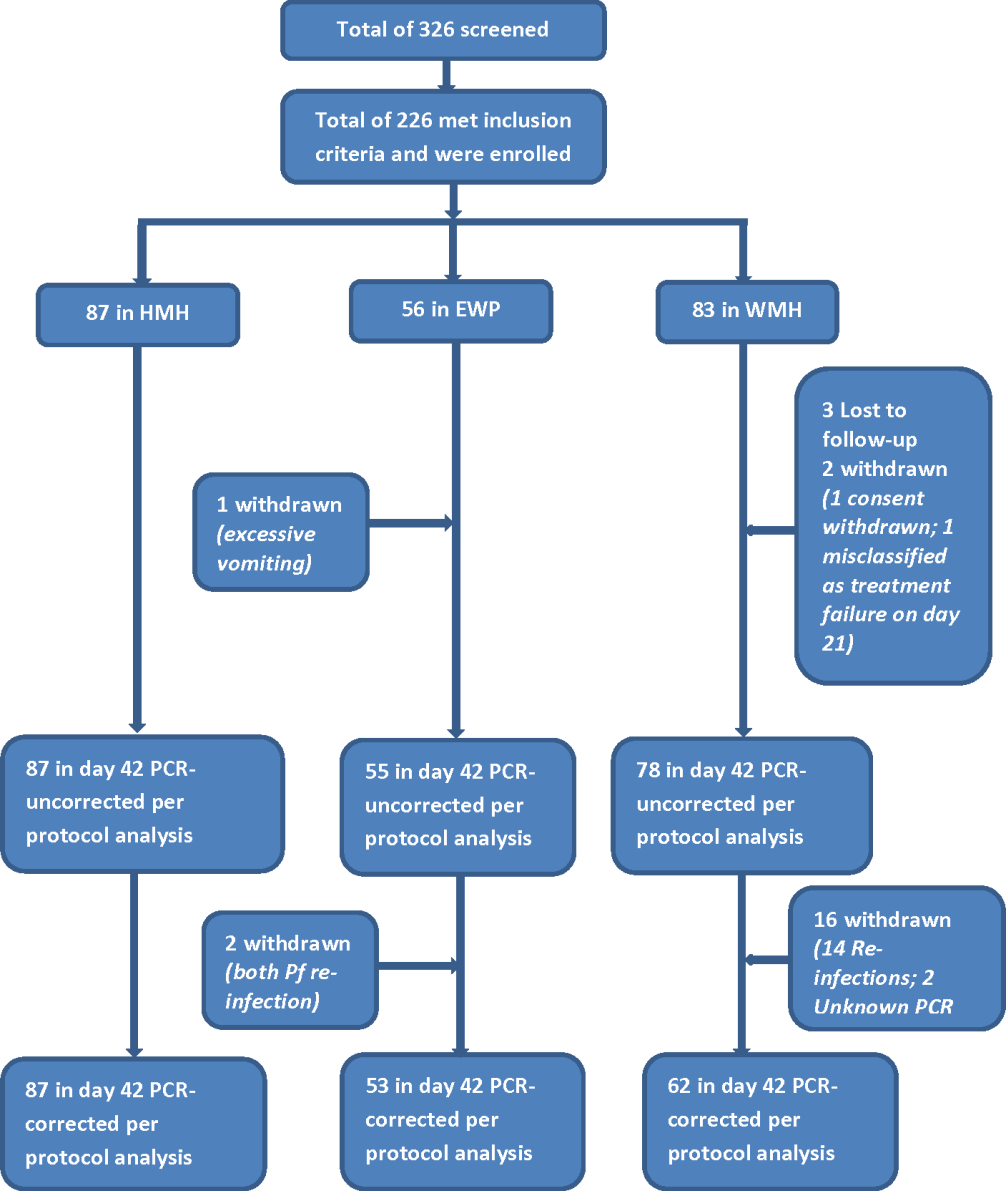
Flow chart showing number of children screened, enrolled, and included in per-protocol analysis. *HMH* Hohoe Municipal Hospital, *EWP* Ewim Polyclinic, *WMH* War Memorial Hospital.

A total of 226 of the 326 children screened met the inclusion criteria and were enrolled into the study in the three sites (**Figure 2**). The proportion of males was higher among participants in EWP (53.6%) and lower among participants in HMH (46.0%) and WMH (48.2%) but not statistically significant (p=0.671) (**Table 2**). The mean age (years) of participants was significantly higher in EWP (5.4 ± 2.4) and WMH (5.4 ± 2.5) compared with HMH (2.2 ± 2.5) (p<0.001). As expected, the mean weight of participants was significantly lower in HMH (11.9 ± 5.8), where a higher proportion of participants (79.3%) were less than 5 years old compared with EWP and WMH (**Table 2**). Mean axillary temperature (°C) was significantly higher in WMH (38.2 ± 0.8) and HMH (38.0 ± 0.6) compared with EWP (37.7 ± 1.1 (p<0.001). Geometric mean parasite density was significantly highest among participants in EWP whilst mean hemoglobin level was significantly higher among participants in WHM (10.9 ± 1.4) compared with HMH (9.9 ± 0.9) and EWP (9.9 ± 1.4) (p<0.001) (**Table 2**). No participant in the three study sites was gametocytemic, and none had a history of previous intake of an antimalarial prior to visiting the clinic.

**Table 2:** Background characteristics of patients enrolled.

Characteristic	Total (N=226)	Sentinel site			p-value	
		HMH (N=87)	EWP (N=56)	NWMH (N=83)		
Gender						
Male	110 (48.7%)	40 (46.0%)	30 (53.6%)	40 (48.2%)		
Female	116 (51.3%)	47 (54.0%)	26 (46.4%)	43 (51.8%)	0.671	
Mean Age in years (SD)	4.2 (2.9)	2.2 (2.5)	5.4 (2.4)	5.4 (2.5)	<0.001	
Age group (years)						
< 5	117 (51.8%)	69 (79.3%)	18 (32.1%)	30 (36.1%)		
5-9	109 (48.2%)	18 (20.7%)	38 (67.9%)	53 (63.9%)	<0.001	
Weight (KG)						
Mean weight (SD)	16.0 (6.6)	11.9 (5.8)	18.0 (5.0)	19.1 (6.1)	<0.001	
Range (min, max)	6.5, 36.0	6.5, 32.0	8.0, 36.0	7.0, 33.0		
Axillary temperature (°C)						
Mean temperature (SD)	38.0 (0.8)	38.0 (0.6)	37.7 (1.1)	38.2 (0.8)	<0.001	
Range (min, max)	35.9, 40.6	36.6, 39.8	35.9, 40.0	37.1, 40.6		
Parasitemia/µL						
Geometric mean	31564	15922	74826	36123	<0.001	
Range (min, max)	1148, 249972	2720, 111391	2966, 249972	1148, 195598		
Hemoglobin level (g/dL)						
Mean (SD)	10.3 (1.3)	9.9 (0.9)	9.9 (1.4)	10.9 (1.4)	<0.001	
Range (min, max)	6.5, 15.2	7.3, 11.6	6.5, 13.5	7.6, 15.2		

SD, Standard Deviation; HMH, Hohoe Municipal Hospital; EWP, Ewim Polyclinic; WMH, War Memorial Hospital.

### 3.2 Primary study outcomes

No day 3 parasitemia was detected in all three sites. Per protocol analyses on day 28 showed no ETF, LCF, and LPF yielding a day-28 DHAP cure rate of 100% in all three sites. Extending the follow-up period to the standard 42-day period showed PCR-uncorrected and PCR-corrected cure rates of 100% for HMH; PCR-uncorrected and PCR-corrected cure rates of 96.4% (95% CI: 86.4-99.4) and 100%, respectively, for EWP; PCR-uncorrected and PCR-corrected cure rates of 71.8% (95% CI: 60.3-81.1) and 90.3% (95% CI: 79.5-96.0), respectively, in WMH (**Table 3**). The national PCR-uncorrected and PCR-corrected cure rates were therefore 89.1% (95% CI: 84.0 – 92.8) and 97.0% (95% CI: 93.4 – 98.8), respectively.

**Table 3:** Per protocol DHAP treatment outcomes.

	Patient Age-group (HMH)			Patient Age-group (EWP)			Patient Age-group (WMH)		
Treatment outcome	< 5 years (N=69)	5-9 years (N=18)	Total (N=87)	< 5 years (N=18)	5-9 years (N=38)	Total (N=56)	< 5 years (N=30)	5-9 years (N=53)	Total (N=83)
PCR uncorrected (Day 42)									
ETF, n (%, 95% CI)	0	0	0	0	0	0	0	0	0
LPF, n (%, 95% CI)	0	0	0	1 (5.9, 0.3 – 30.8)	1 (2.6, 0.1-15.4)	2 (3.6, 0.6-13.6)	6 (21.4, 9.0-41.5)	14 (28.0, 16.7-42.7)	20 (25.6, 16.7-37.0)
LCF, n (%, 95% CI)	0	0	0	0	0	0	2 (7.1, 1.3-25.0)	0	2 (2.6, 0.5-9.8)
ACPR, n (%, 95% CI)	69 (100, 93.4-)	18 (100, 78.1-)	87 (100, 94.7-)	16 (94.1, 69.2-99.7)	37 (97.4, 84.6-99.9)	53 (96.4, 86.4-99.4)	20 (71.4, 51.1-86.1)	36 (72, 57.3-83.3)	56 (71.8, 60.3-81.1)
Total per protocol	69	18	87	17	38	55	28	50	78
Lost/withdrawn, n (%, 95% CI)	0	0	0	1 (5.6, 0.3-29.4)	0	1 (1.8, 0.1-10.8)	2 (6.7, 1.2-23.5)	3 (5.7, 1.5-16.6)	5 (6.0, 2.2-14.1)
PCR corrected (Day 42)									
ETF, n (%, 95% CI)	0	0	0	0	0	0	0	0	0
LPF, n (%, 95% CI)	0	0	0	0	0	0	2 (9.1, 1.6-30.6)	4 (10.0, 3.3-24.6)	6 (9.7, 4.0-20.5)
LCF, n (%, 95% CI)	0	0	0	0	0	0	0	0	0
ACPR, n (%, 95% CI)	69 (100, 93.4-)	18 (100, 78.1-)	87 (100, 94.7-)	16 (100, 75.9-)	37 (100, 88.3-)	53 (100, 91.6-)	20 (90.9, 69.4-98.4)	36 (90.0, 75.4-96.8)	56 (90.3, 79.5-96.0)
Total per protocol	69	18	87	16	37	53	22	40	62
Lost/withdrawn, n (%, 95% CI)	0	0	0	2 (11.1, 1.9-36.1)	1 (2.6, 0.1-15.4)	3 (5.4, 1.4-15.8)	8 (36.4, 18.0-59.2)	13 (32.5, 19.1-49.2)	21 (33.9, 22.7-47.1)


HMH, Hohoe Municipal Hospital; EWP, Ewim Polyclinic; WMH, War Memorial Hospital; ETF, Early Treatment Failure; LPF, Late Parasitological Failure; LCF, Late Clinical Failure; ACPR, Adequate Clinical and Parasitological Response.

**Figure 3:**
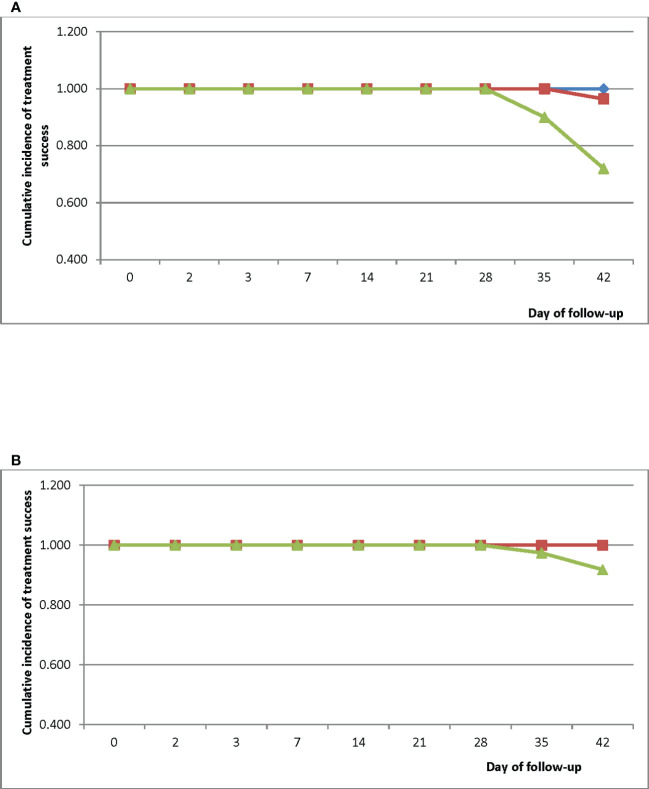
**(A)** PCR-uncorrected Kaplan-Meier survival curve for children treated with DHAP in three sentinel sites in Ghana. Blue line represents Hohoe Municipal Hospital (HMH); red line represents Ewim Polyclinic (EWP); and green line represents War Memorial Hospital (WMH). **(B)** PCR-corrected Kaplan-Meier survival curve for children treated with DHAP in three sentinel sites in Ghana. Blue line represents Hohoe Municipal Hospital (HMH); red line represents Ewim Polyclinic (EWP); and green line represents War Memorial Hospital (WMH).

Kaplan-Meier survival analyses showed a PCR-uncorrected cumulative treatment success incidence of 1.000 for all three sites between Day 0 and Day 28 (**Figure 3A**). Cumulative success incidence in HMH remained 1.000 on Days 35 and 42 whilst incidence in EWP remained 1.000 on Day 35 and dropped to 0.960 (95% CI: 0.859-0.992) on Day 42. Cumulative PCR-uncorrected treatment success incidence in WMH dropped to 0.900 (95% CI: 0.806-0.953) on Day 35 and further dropped to 0.720 (95% CI: 0.605-0.813) on Day 42. PCR-corrected cumulative treatment success incidence in HMH was 1.000 on Day 42 whilst incidence in WMH was 0.974 (95% CI: 0.887-0.997) on Day 35 and 0.918 (95% CI: 0.813-0.969) on Day 42 (**Figure 3B**) (WHO, 2009).

### 3.3 Secondary study outcomes

**Figure 4:**
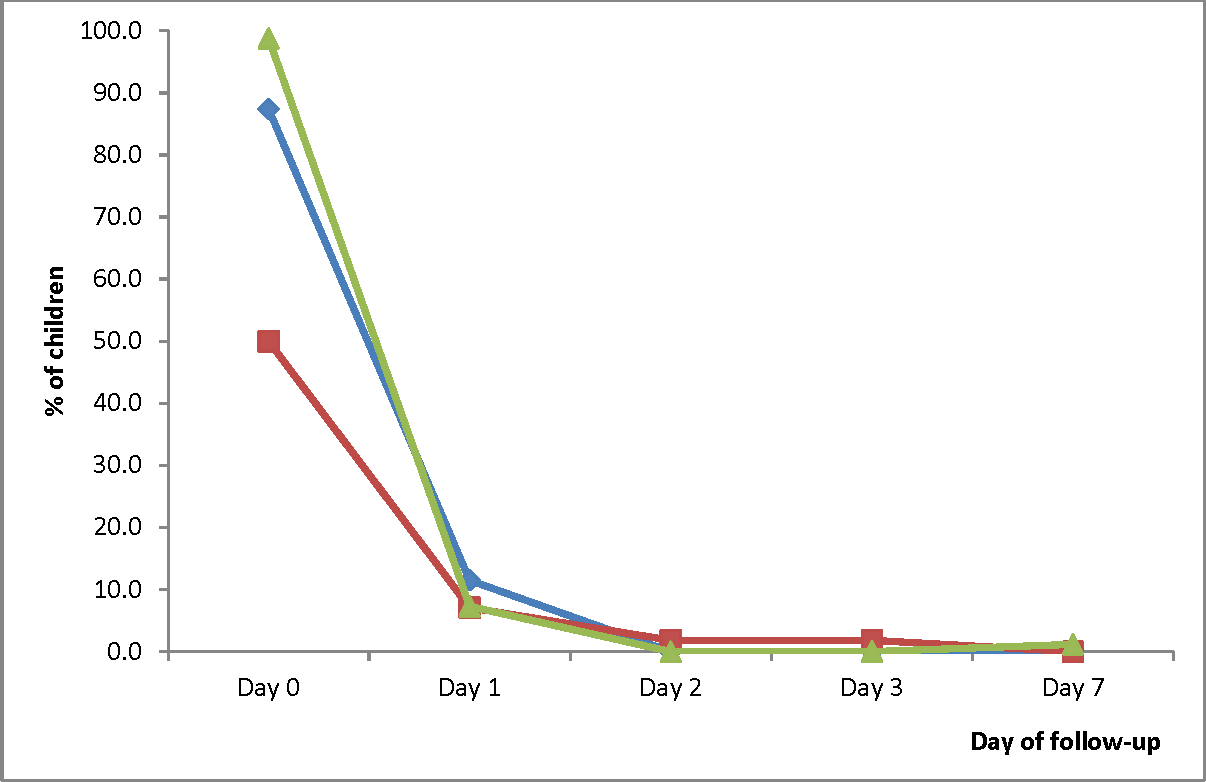
Proportion of children with measured fever (axillary temperature ≥ 37.5°C) during the first week of follow-up. Blue line represents Hohoe Municipal Hospital (HMH); red line represents Ewim Polyclinic (EWP); and green line represents War Memorial Hospital (WMH).

The prevalence of measured fever (axillary temperature ≥ 37.5°C) significantly decreased from Day 0 to Day 1 in all three sites: 87.4% (95% CI: 78.1-93.3) to 11.5% (95% CI: 6.0-20.6) in HMH; 50.0% (95% CI: 36.5-63.5) to 7.1% (95% CI: 2.3-18.1) in EWP; and 98.8% (95% CI: 93.1-99.9) to 7.3% (95% CI: 3.0-15.8) in WMH. On Day 2, no child in HMH and WMH had axillary temperature ≥ 37.5°C (**Figure 4**). The only child in EWP with axillary temperature ≥ 37.5°C on Day 2 was aparasitemic. On Day 3, no child in HMH and WMH had axillary temperature ≥ 37.5°C. The only child in EWP with axillary temperature ≥ 37.5°C on Day 3 was aparasitemic. On Day 7, no child in HMH and EWP had axillary temperature ≥ 37.5°C. The only child in WMH with axillary temperature ≥ 37.5°C on day 7 was aparasitemic.

**Figure 5:**
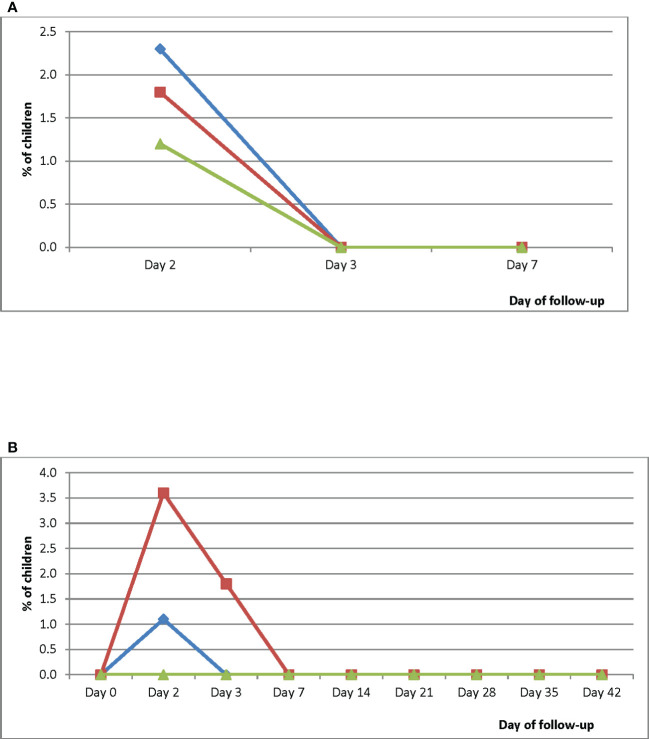
**(A)** Proportion of children with parasitemia during the first week of follow-up. Blue line represents Hohoe Municipal Hospital (HMH); red line represents Ewim Polyclinic (EWP); and green line represents War Memorial Hospital (WMH). **(B)** Proportion of children with gametocytemia during the 42-day follow-up period. Blue line represents Hohoe Municipal Hospital (HMH); red line represents Ewim Polyclinic (EWP); and green line represents War Memorial Hospital (WMH).

Parasite prevalence on Day 2 ranged between 1.2% (95% CI: 0.1-7.5) in WMH and 2.3% (95% CI: 0.4-8.8) in HMH. No child was parasitemic on Day 3 and Day 7 (**Figure 5A**). Gametocytemia was prevalent in HMH on Day 2 only (1.1%; 95% CI: 0.6-2.1) and in EWP on Day 2 (3.6%; 95% CI: 0.6-13.6) and Day 3 (1.8%; 95% CI: 0.1-11.0). Gametocytemia was not prevalent in WMH during the follow-up period (**Figure 5B**).

Mean haemoglobin levels on days 28 and 42 were significantly higher than pre-treatment levels in all three sites (**Table 4**). The mean changes between day 28 and day 42 were significant in HMH and EWP (p<0.001 and p=0.006, respectively) but not WMH (p=0.075).

**Table 4:** Changes in mean hemoglobin levels following treatment with DHAP.

Site	Mean Hb (sd)			p-value
	Day 0	Day 28	Day 42	
HMH	9.9 (0.9) N=87	11.5 (0.4) N=87	11.8 (0.3) N=87	< 0.001
EWP	9.9 (1.4) N=56	10.9 (0.7) N=55	11.3 (0.8) N=55	< 0.001
WMH	10.9 (1.4) N=83	11.3 (1.0) N=78	11.6 (1.1) N=78	<0.001

HMH, Hohoe Municipal Hospital; EWP, Ewim Polyclinic; WMH, War Memorial Hospital.

**Figure 6:**
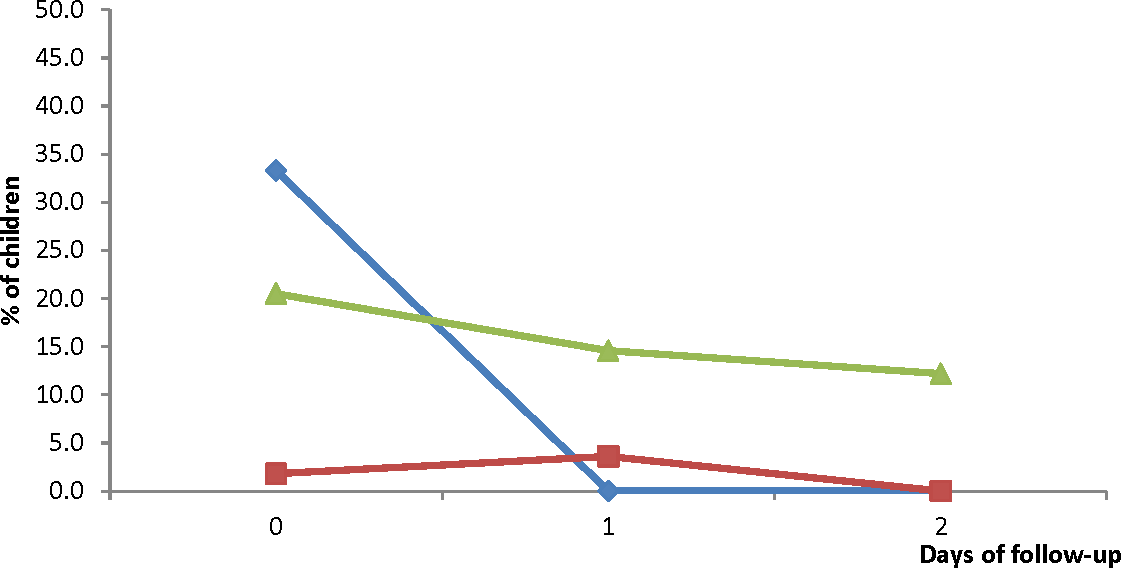
Proportion of children vomiting during the three days of treatment with DHAP. Blue line represents Hohoe Municipal Hospital (HMH); red line represents Ewim Polyclinic (EWP); and green line represents War Memorial Hospital (WMH).

Vomiting was the main adverse event reported. Prevalence of vomiting on day 0 was significantly highest in HMH compared with EWP and WMH (33.3%; 95% CI: 23.8-44.4 *vs* 1.8%; 95% CI: 0.1-10.8 *vs* 20.5%; 95% CI: 12.7-31.0; p<0.001) but significantly decreased to 0% on days 1 and 2 (**Figure 6**). The changes observed in EWP (3.6%; 95% CI: 0.6-13.4 on day 1 and 0% on day 2) were not significant (p=0.421). Likewise, the changes observed in WMH (14.6%; 95% CI: 8.1-24.6 on day 1 and 12.2%; 95% CI: 6.3-21.7 on day 2) were not significant (p=0.322) (**Figure 6**). Even though prevalence of vomiting in WMH was over 10% on days 1 and 2, there was no significant difference in treatment failure between those who vomited and those who did not (22.2% *vs* 33.3%; p=0.404 for PCR-uncorrected failures and 6.7% *vs* 12.5%; p=0.732 for PCR-corrected failures).

## 4 Discussions

The therapeutic efficacy of Ghana’s second-line antimalarial for the treatment of uncomplicated malaria, DHAP, was studied in three sentinel sites representing the three main ecological zones of the country in 2020. The study was to generate DHAP efficacy data to serve as baseline for subsequent studies using the standard 42-day follow-up schedule.

All children examined on day 3, the day used to assess artemisinin partial resistance (WHO, 2020), were aparasitemic suggesting an adequate response of parasites to dihydroartemisinin, and supporting the observation that artemisinin resistance is currently not a problem in Ghana (Abuaku et al., 2021).

The study showed day-28 PCR-uncorrected efficacy levels of 100% in all three sites. This finding of 100% PCR-uncorrected cure rate in all sites representing the three main ecological zones of Ghana has not been observed for Ghana’s first-line ACTs (AL and ASAQ) studied over the years. In 2005, ASAQ was studied in EWP and WMH with PCR uncorrected cure rates of 96.1% (95% CI: 88.0 – 99.0) in EWP and 98.7% (95% CI: 92.1 – 99.9) in WMH (Koram et al., 2008). In 2010, AL was studied in EWP and WMH with PCR-uncorrected cure rates of 79.0% (95% CI: 62.2 – 89.9) in EWP and 83.9% (95% CI: 71.2 – 92.0) in WMH (Abuaku et al., 2012). In 2013, ASAQ and AL were studied in sites in the forest zone (including HMH) and savannah zone (including WMH) with PCR-uncorrected ASAQ cure rates of 98.0% (95% CI: 88.0 – 99.9) in the forest zone and 97.3% (95% CI: 89.8 – 99.5) in the savannah zone and AL cure rates of 85.1% (95% CI: 77.2 – 90.7) in the forest zone and 56.3% (95% CI: 37.9 – 73.2) in the savannah zone (Abuaku et al., 2016). A study conducted in EWP in 2014 showed PCR-uncorrected ASAQ cure rate of 93.6% (95% CI: 86.1 – 97.4) (Abuaku et al., 2017). In 2015, PCR-uncorrected ASAQ cure rates were reported to be 98.2% (95% CI: 89.0 – 99.9) in WMH and 93.5% (83.4 – 97.9) in EWP. In the same year PCR-uncorrected AL cure rates were reported to be 91.4% (95% CI: 80.3 – 96.8) in WMH and 85.7% (95% CI: 74.1 – 92.9) in EWP (Abuaku et al., 2019). These results suggest some superiority of DHAP over ASAQ and AL in terms of chemoprophylaxis, and supports the observation of longer prophylactic effect of DHAP on new malaria infections (Karema et al., 2006; Myint et al., 2007; Price and Douglas, 2009; WHO, 2010; Akpaloo and Purssell, 2014; Onyamboko et al., 2014; Zani et al., 2014; Uwimana et al., 2019; Assefa et al., 2021).

PCR-corrected cure rates for all three sites on day 42 were over 90% (failure rates below WHO’s threshold of 10% for partner drug resistance) (WHO, 2020), and therefore supports the inclusion of DHAP in the treatment policy for Ghana (MOH, 2020). High efficacy of DHAP has also been reported in other sub-Saharan African countries (Marwa et al., 2022) including Nigeria (Ebenebe et al., 2018), Sierra Leone (Smith et al., 2018), Mali (Dama et al., 2018), Guinea-Bissau (Ursing et al., 2016), Kenya (Westercamp et al., 2022), Tanzania (Mandara et al., 2019), Uganda (Ebong et al., 2021), Somalia (Warsame et al., 2019), Rwanda (Uwimana et al., 2019), and Angola (Davlantes et al., 2018). This notwithstanding PCR-corrected cure rate for WMH was significantly lower than HMH and EWP suggesting possible lower parasite susceptibility to piperaquine in WMH. To better explain differences like this, parallel pharmacokinetic studies have been incorporated into subsequent efficacy studies in Ghana.

As with other ACTs, DHAP achieved rapid parasite and fever clearance in all three sites. Parasite prevalence declined from 100% on day 0 to 1.2-2.3% on day 2 whilst prevalence of measured fever declined from 50.0-98.8% on day 0 to 7.1-11.5% on day 1. Also, mean hemoglobin levels significantly increased in all three sites after treatment with DHAP. These observations compare well with findings from previous Ghanaian studies (Abuaku et al., 2019; Abuaku et al., 2021) and studies from other African countries (Dama et al., 2018; Davlantes et al., 2018; Mandara et al., 2018; Raobela et al., 2018; Roth et al., 2018) demonstrating the effectiveness of ACTs in parasite and fever clearance as well as improving hemoglobin levels of uncomplicated malaria patients.

## 5 Conclusion

Findings of this study suggest that DHAP is highly efficacious in Ghana achieving over 90% cure rates in the treatment of uncomplicated malaria, and has the advantage of a longer prophylactic effect over new infections compared with ASAQ and AL. There is also no evidence of artemisinin partial resistance as per WHO definition (≥ 10% of patients with asexual parasites on day 3 post-treatment). Subsequent DHAP efficacy studies, using current data as baseline, are critical in monitoring the usefulness of DHAP as second line ACT for uncomplicated malaria in Ghana.

## Data availability statement

The raw data supporting the conclusions of this article will be made available by the authors, without undue reservation.

## Ethics statement

The studies involving human participants were reviewed and approved by Institutional Review Board, Noguchi Memorial Institute for Medical Research, University of Ghana. Written informed consent to participate in this study was provided by the participants’ legal guardian/next of kin.

## Author contributions

BA, PB, NP, AA, ND-Q, NQ, EA, FO-A, KM and KK participated in the design and supervision of the study. ND-Q, SM and NQ performed PCR genotyping to distinguish re-infections from recrudescence. BA and EA performed the statistical analyses. BA drafted the manuscript. and shared with authors. All authors contributed to the article and approved the submitted version.
